# Lacrimal Gland Fistula following Severe Head Trauma

**DOI:** 10.1155/2015/534985

**Published:** 2015-03-05

**Authors:** Cemil Demir, Ibrahim Toprak, Sukru Gungen, Alp Arslan

**Affiliations:** ^1^Department of Ophthalmology, Servergazi State Hospital, 20275 Denizli, Turkey; ^2^Department of Neurosurgery, Servergazi State Hospital, 20275 Denizli, Turkey

## Abstract

We aim to present a unique case with discharging lacrimal gland fistula secondary to severe head trauma by an animal. A 9-year-old girl presented with serous fluid discharge from a cutaneous fistula in the left orbital region. The patient had history of surgery for traumatic frontal bone fracture and skin laceration in the superior orbital rim three weeks earlier. She underwent a complete ophthalmological examination and there was no anterior segment or fundus pathology. The orifice of the fistula was detected in mediolateral part of the left superior orbital rim and fluid secretion was increasing with irritation of the left eye. Neurosurgical complications were excluded and radiological assessment was nonremarkable. The patient's legal representatives were informed and lacrimal gland fistulectomy was planned. However, the fistula was self-closed one week after initial ophthalmological examination, and the patient had no symptoms. In conclusion, traumatic injuries of superior orbital region should be carefully evaluated and wounds should be well closed to prevent consecutive lacrimal gland fistula.

## 1. Introduction 

The lacrimal gland is a bilobular, tubuloacinar gland located in the lacrimal fossa of the frontal bone. It contains multiple ducts that secrete the aqueous portion of the tear film [1, 2]. In the current literature, a number of studies reported lacrimal gland injuries and fistulas [1–4]. In 1980, Putterman firstly presented a child with epiphora secondary to lacrimal gland fistula [1]. In recent years, formation of lacrimal gland fistula following eyelid surgery was reported in several studies [3, 4].

In the current report, we present clinical characteristics of a child with lacrimal gland fistula following severe head trauma by a goat, which is a very rare condition.

## 2. Case Report 

A 9-year-old girl was referred to ophthalmology department for clear fluid discharge from a cutaneous fistula for one week. She had operation history for frontal bone fracture (without meningeal or cerebral involvement) secondary to severe head trauma by a goat three weeks earlier. From the hospital records, the initial trauma had also caused skin laceration in the left superior orbital rim and it had been sutured. However, suture rejection and wound dehiscence had been observed five days after the initial trauma. A plastic surgeon had examined the patient and the wound had been left to secondary healing.

**Figure 1:**
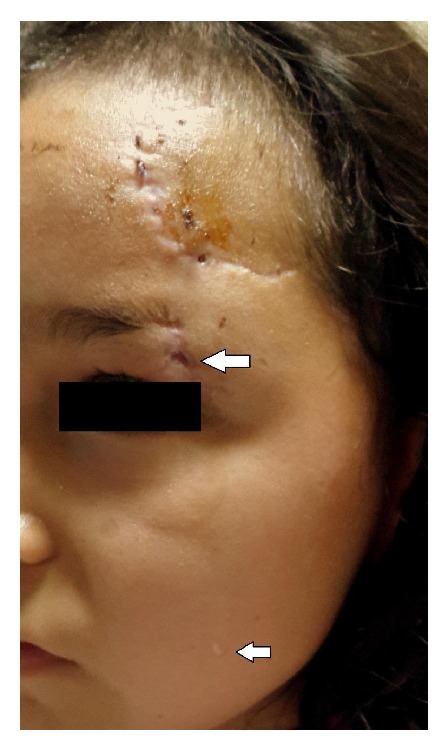
The photograph was captured immediately after irritation of the left eye with penlight. The big white arrow shows the orifice of the lacrimal gland fistula in the left superior orbital rim and small white arrow points out a teardrop, which was leaked from the fistula.

The patient underwent a detailed ophthalmological examination. There was no restriction in eye movements. Visual acuity was 10/10 (Snellen chart) in both eyes; Schirmer's test, anterior segment, and fundus examinations were normal. However, the orifice of the discharging cutaneous fistula was observed in the mediolateral portion of the superior orbital rim (eyebrow), which had been previously sutured, and we detected an increase in serous secretion with emotional stimuli and irritation of the left eye (Figure 1). In the left frontal region, “C” shaped cicatrix of the skin incision (traumatic and surgical) was noted (Figure 1). Orbital computerized tomography and magnetic resonance imaging findings were nonspecific. An experienced neurosurgeon examined the patient. There was no active cranial pathology and serous secretion from the fistula was not considered as cerebrospinal fluid (CSF).

Assuming that lacrimal gland fistula was developed during the postoperative course, the patient and legal representatives were informed and left lacrimal gland fistulectomy was planned. However, during the preoperative preparation, the fistula was self-healed and symptoms disappeared. The patient is currently under our follow-up.

## 3. Discussion 

Lacrimal gland is located in superior-temporal orbital region and can be injured by head trauma. A few numbers of studies reported lacrimal gland fistulas [1–4]. The most common causes are congenital and iatrogenic (postoperative). Putterman firstly described lacrimal gland fistula in a child, and epiphora was the major symptom [1]. It was not concluded whether it was congenital or traumatic [1]. Furthermore, congenital misdirected lacrimal gland ductules were described by Cogen et al. [2]. Iatrogenic lacrimal gland injury is the other most cited cause of the lacrimal gland fistula. Kashkouli et al. [3] and Ahn et al. [4] reported formation of lacrimal gland fistula following upper eyelid surgery.

In the present case report, we described a unique case with discharging lacrimal gland fistula following severe frontal trauma by an animal. The frontal bone fracture in the patient brought us to suspect whether serous discharge was CSF, whereas absence of prior meningeal injury and neurosurgical consultation excluded the CSF leakage. Moreover, the increase in clear fluid discharge with emotional stimuli and irritation of the eye supported our diagnosis.

In conclusion, it should be kept in mind that superior orbital and frontal trauma could lead to lacrimal gland injury and secondary fistula. Skin wounds in this region should be carefully explored and well closed.
